# Diminished learning rates in mnemonic discrimination of scenes but not objects are associated with beta‐amyloid positivity

**DOI:** 10.1002/alz.089785

**Published:** 2025-01-03

**Authors:** Sarah E. Polk, Helena M. Gellersen, Yannik Lottes, Wenzel Glanz, David Berron

**Affiliations:** ^1^ German Center for Neurodegenerative Diseases (DZNE), Magdeburg Germany; ^2^ Institute of Psychology, Otto‐von‐Guericke University, Magdeburg Germany; ^3^ Clinical Memory Research Unit, Lund University, Lund Sweden

## Abstract

**Background:**

The advent of remote and unsupervised digital cognitive assessment facilitates the use of frequent assessments only days apart to examine learning rates. Learning rates have been shown to differentiate patients with Mild Cognitive Impairment (MCI) and preclinical Alzheimer’s disease (AD) from healthy individuals across a variety of cognitive measures. Here, we investigated whether impairments in learning rates in a mnemonic discrimination task were domain specific, as object and scene memory have been found to rely on anatomically distinct neural pathways that are differentially affected by AD.

**Methods:**

We examined learning rates over two weeks in the Mnemonic Discrimination Task for Objects and Scenes (MDT‐OS) in N = 54 older adults (age = 67.1 ± 5.68) recruited through a memory clinic who were clinically cognitively unimpaired (CU, n = 44, n_Aβ–_ = 28, n_Aβ+_ = 6) or had MCI (n = 10, n_Aβ–_ = 3, n_Aβ+_ = 7). Five identical stimuli sets were completed at three‐day intervals on participants’ own smartphone or tablet.

**Results:**

Using linear mixed modeling, we found significant cognitive status‐by‐time interactions indicating that the MCI group showed reduced learning compared to the CU group in the *Scene* condition, though not the *Object* condition. However, a cognitive status‐by‐time‐by‐condition model indicated no significant effect of condition. We found no significant Aβ status‐by‐time interaction in either condition. However, an Aβ status‐by‐time‐by‐condition model revealed diminished learning in the *Scene* condition compared to the *Object* condition for the Aβ+ group but not the Aβ– group. In a sub‐group analysis including only CU individuals, this three‐way interaction effect remained significant.

**Conclusion:**

The current results suggest that individuals with MCI show diminished learning rates in mnemonic discrimination of scenes, compared to CU individuals. We identified ceiling effects in the *Object* condition, which may have contributed to a null finding. Additionally, we found that Aβ positivity was associated with reduced learning of scenes but not objects, both in the overall sample and in individuals with no clinical indication of cognitive impairment. In conclusion, learning rates in the MDT‐OS may be a sensitive marker of AD in individuals who do not yet meet the clinical threshold for cognitive impairment.

